# First-line surgery in prolactinomas: lessons from a long-term follow-up study in a tertiary referral center

**DOI:** 10.1007/s40618-021-01569-6

**Published:** 2021-04-13

**Authors:** L. Andereggen, J. Frey, R. H. Andres, M. M. Luedi, M. El-Koussy, H. R. Widmer, J. Beck, L. Mariani, R. W. Seiler, E. Christ

**Affiliations:** 1 Department of Neurosurgery, Neurocenter and Regenerative Neuroscience Cluster, Inselspital, Bern University Hospital, University of Bern, Freiburgstrasse, 3010 Bern, Switzerland; 2 Department of Neurosurgery, Kantonsspital Aarau, Aarau, Switzerland; 3 Department of Endocrinology, Diabetes, Nutrition and Metabolism, Inselspital, Bern University Hospital, University of Bern, Bern, Switzerland; 4 Department of Gynecology and Obstetrics, Kantonsspital Lucerne, Lucerne, Switzerland; 5 Department of Anaesthesiology and Pain Medicine, Inselspital, Bern University Hospital, University of Bern, Bern, Switzerland; 6 Institute of Diagnostic and Interventional Neuroradiology, Inselspital, Bern University Hospital, University of Bern, Bern, Switzerland; 7 Department of Neurosurgery, Medical Center, University of Freiburg, Freiburg, Germany; 8 Department of Neurosurgery, University Hospital of Basel, Basel, Switzerland; 9 Department of Endocrinology, Diabetes and Metabolism, University Hospital of Basel, Basel, Switzerland

**Keywords:** Dopamine agonists, Long-term outcome, Macroadenoma, Microadenoma, Knosp grading, Primary surgical therapy, Prolactinoma

## Abstract

**Context:**

Although consensus guidelines recommend dopamine agonists (DAs) as the first-line approach in prolactinomas, some patients may opt instead for upfront surgery, with the goal of minimizing the need for continuation of DAs over the long term. While this approach can be recommended in selected patients with a microprolactinoma, the indication for upfront surgery in macroprolactinomas remains controversial, with limited long-term data in large cohorts. We aimed at elucidating whether first-line surgery is equally safe and effective for patients with micro- or macroprolactinomas not extending beyond the median carotid line (i.e., Knosp grade ≤ 1).

**Methodology:**

Retrospective study of patients with prolactinomas Knosp grade ≤ 1 treated with upfront surgery. The primary endpoint was patients’ dependence on DAs at last follow-up. The secondary endpoint was postoperative complications. Independent risk factors for long-term dependence on DAs were analyzed.

**Results:**

A microadenoma was noted in 45 patients (52%) and a macroadenoma in 41 (48%), with 17 (20%) harboring a Knosp grade 1 prolactinoma. Median follow-up was 80 months. First-line surgery resulted in long-term remission in 31 patients (72%) with a microprolactinoma and in 18 patients (45%) with a macroprolactinoma (*p* = 0.02). DA therapy was ultimately required in 11 patients (24%) with microadenomas vs. 20 (49%) with macroadenomas (*p* = 0.03). As for the latter, DA was required in 13 patients (76%) with Knosp grade 1 macroadenomas vs. 7 patients (29%) with Knosp grade 0 macroadenomas (p = 0.004). There was no mortality, and morbidity was minimal. Knosp grade 1 prolactinomas (OR 7.3, 95% CI 1.4–37.7, *p* = 0.02) but not adenoma size (i.e., macroprolactinomas) were an independent predictor of long-term dependence on DAs.

**Conclusions:**

First-line surgery in patients with microprolactinomas or macroprolactinomas Knosp grade 0 resulted in a good chance of non-dependency on DA therapy. However, in patients with prolactinomas Knosp grade 1, first-line surgery cannot be recommended, as adjuvant DA therapy after surgery is required in the majority of them over the long term.

## Introduction

While consensus guidelines recommend dopamine agonists (DAs) as the first-line approach in the treatment of prolactinomas, [1–4] surgery is primarily indicated in patients who are resistant to or intolerant of DAs, in cases of cystic adenomas, or intratumoral hemorrhage with persisting visual disturbances [5–11]. Thus, most surgical series report the results of a second-line approach in patients with evidence of resistance or intolerance to DAs [5, 12].

In 2006, the Pituitary Society revised its guidelines to include surgery in dedicated centers with experienced surgeons as a possible first-line approach for prolactinomas when it is the patient’s preference, rather than long-term DA therapy [13]. This approach has increasingly emerged given that DAs are required over the long-term in up to 80% of patients. [14] DAs have been associated with side effects such as nausea, dizziness, and postural hypotension; however, they are present in only a minority of patients [15]. Although side effects of cabergoline were recorded in 68% of women in a large cohort with hyperprolactinemic amenorrhea, only 3% of them ultimately had to discontinue drug therapy due to intolerance.[16] Also, cabergoline-associated valvulopathy is uncommon, [17, 18] and its clinical significance remains unclear [19]. In a recent cohort study, no association was reported between a clinically significant valvulopathy and low-dose cabergoline therapy [20]. On rare occasions, personality changes associated with DAs have been reported, including gambling, hypersexuality and compulsive shopping [21–23]. It is possible that patients don’t mention these effects due to feelings of shame, with potential detrimental psychosocial consequences [24]. Importantly, though, the low prevalence of these side effects should not cast doubt on the well-defined medical treatment approach to prolactinomas [25, 26]. The renewed acceptance of surgical treatment of prolactinomas, however, primarily applies to patients with a microprolactinoma, in whom a short-term cure rate of about 90% can be anticipated [27–31]. However, the indication for upfront surgery in macroprolactinomas remains controversial, and long-term data on patients in large cohorts is limited [8, 32–34].

Our purpose was to elucidate whether first-line surgery is equally safe and effective for patients with microprolactinomas or macroprolactinomas not extending beyond the median carotid line (Knosp grade ≤ 1). In particular, we aimed at investigating whether tumor characteristics at diagnosis have an impact on the control of hyperprolactinemia and long-term dependence on DAs.

## Patients and methods

### Study design

We conducted a retrospective study reviewing data from prolactinoma patients stored in our institutional database. The records were prospectively maintained from January 1996 to December 2015. All consecutive patients in whom first-line surgery was performed for the treatment of either micro- or macroprolactinomas were analyzed. The Human Research Ethics Committee of Bern (Kantonale Ethikkommission KEK Bern, Bern, Switzerland) approved the project (KEK n° 10-10-2006 and 8-11-2006).

### Preoperative assessment

Diagnosis was based on preoperative clinical and biochemical assessment as well as a standard protocol for pituitary magnetic resonance imaging (MRI; see below). Types of DA-agonist therapy and maximal doses were noted (e.g., bromocriptine, quinagolide, cabergoline).

#### Clinical assessment

Baseline characteristics included patients’ age, sex, body mass index (BMI), and clinical reason for presentation (i.e., headache, visual deficits).

#### Biochemical assessment

PRL levels, including the immunoradiometric PRL assay (IRMA), which uses serum dilution to overcome the high-dose PRL hook effect, [35] were assessed. The presence of macroprolactin was routinely assessed [36]. Upper limits of PRL levels were 20 ng/mL [37]. As for pituitary axis deficits, partial hypopituitarism was defined as impaired secretion of one or more pituitary hormones. Secondary adrenal insufficiency was characterized by the presence of low cortisol (< 50 nmol/L) levels in the serum, or normal cortisol but inadequate responses to the adrenocorticotropin (ACTH) stimulation test or insulin tolerance test. The diagnosis of secondary hypothyroidism was based on a finding of low-normal thyroid-stimulating hormone (TSH) levels and low free thyroxin (FT4) level. A gonadotropin deficiency or central hypogonadism was considered in the case of low-normal levels of gonadotropins in parallel with low estradiol/testosterone levels.

#### MRI evaluation

MRI was performed on a 1.5- or 3-T system including a Proton/T2-weighted whole-brain study with unenhanced, contrast-enhanced, dynamic contrast-enhanced and post contrast-enhanced overlapping studies in the axial, sagittal and coronal planes over the sellar region [38, 39]. A tumor with a diameter of 1–10 mm was defined as a microadenoma and > 10 mm as a macroadenoma. Knosp classification was used to describe invasiveness of the cavernous sinus [40, 41]. The gold standard for diagnosis was immunohistochemical confirmation with a PRL antibody as an immunohistochemical marker according to the WHO classification of neuroendocrine tumors [42].

### Indication for surgery

For all patients, the indication for surgery was discussed at an interdisciplinary pituitary tumor board meeting, with consensus tailored to preventing patients from becoming dependent on DA therapy over the long term. The indication for upfront surgery was further discussed with the patient and based on patients’ preference for surgical treatment rather than long-term DA therapy. Surgery was considered both for microprolactinomas and macroprolactinomas not extending beyond the medial carotid line (i.e., Knosp grade ≤ 1). Because health insurance in Switzerland covers both medical and surgical therapy of all its residents, treatment decisions are not based on financial considerations [43].

Pituitary surgery was performed by three experienced neurosurgeons (RWS, LM, JB) using a transseptal, transsphenoidal microsurgical approach with sellar reconstruction, as previously described [44].

### Inclusion and exclusion criteria

**Fig. 1:**
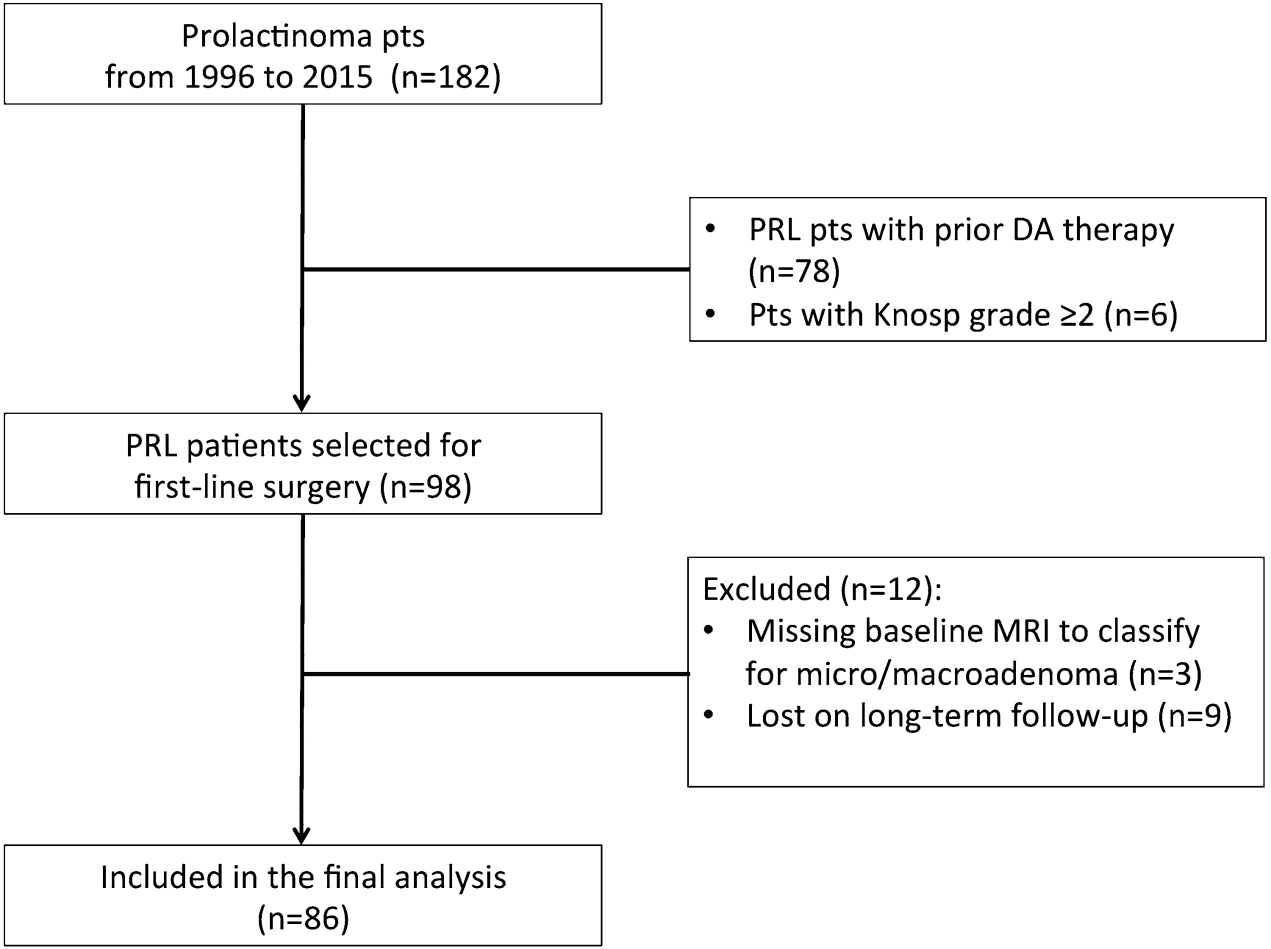
Flow chart of patient selection process. Out of 182 patients with a prolactinoma, first-line surgery was performed in 98 patients, with 86 patients included in the final analysis given the presence of long-term follow-up data

A flow chart of the patient selection process is depicted in Fig. 1. Patients who had previously received DAs were excluded from the study (*n* = 78). Patients with cavernous sinus infiltration (i.e., Knosp grade ≥ 2) were not considered for a primary surgical approach (*n* = 6). Furthermore, twelve patients were excluded from this study given the missing baseline MRI data for classification as a microadenoma or a macroadenoma (*n* = 3), and the missing data on their long-term follow-up (*n* = 9).

### Postoperative and long-term assessment

Early follow-up took place three months after pituitary surgery. In patients with elevated PRL levels (> 20 µg/L) at three months, DA therapy was initiated (e.g., bromocriptine, quinagolide, and mainly cabergoline) [45] In patients with marginally increased prolactin levels above the normal range but lacking clinical symptoms, DA therapy was not initiated, and prolactin levels were controlled during routine follow-up. Late follow-up was defined as the last documented visit to the endocrine outpatient clinic of the Bern University Hospital. DAs were tapered 24 months after initiation of medical therapy if PRL levels had normalized [46, 47]. There was no routine follow-up to control the tumor size by means of sellar MRI.[48, 49] Patients were considered to be in remission if the PRL level was < 20 µg/L at follow-up.

### Statistical analyses

Data were analyzed using IBM SPSS statistical software Version 24.0 (IBM Corp., New York, NY, USA) and GraphPad Prism (V7.04 software, San Diego, CA, USA). Continuous variables were examined for homogeneity of variance and are expressed as mean ± SD unless otherwise noted. Serum PRL levels are presented as median values and interquartile range (IQR, 25th to 75th percentile). Categorical variables are given as numbers and percentages. For comparisons of means between groups (i.e., patients with micro- and macroadenomas), Student’s *t*-test was used for normally distributed data, and the Mann–Whitney test for nonparametric data. The Wilcoxon signed-rank test was used to evaluate paired differences in PRL levels before and after treatment. Categorical variables were compared using Pearson’s chi-square test or Fisher’s exact test, as appropriate. The Kaplan–Meier method was used to analyze recurrence-free intervals during follow-up, and the significance was calculated using the log-rank (Mantel–Cox) test. Odds ratios (ORs) and 95% confidence intervals (CIs) of independent factors for early negative outcome (i.e. postoperative PRL levels > 20 μg/L) were analyzed by univariable and multivariable logistic regression. We assessed the proportion of patients with long-term dependence on DAs and performed time-dependent multivariable regression analysis to calculate hazard ratios (HR) for potential risk factors. The variables tested were: age at diagnosis, sex, headache at presentation, hypopituitarism at diagnosis, BMI (kg/m^2^), initial PRL levels, adenoma size, and Knosp classification. The multivariable regression analysis included all dependent risk factors in the univariable regression with a *p* value ≤ 0.05. Baseline PRL values were log transformed before being imputed in the regression analysis, as data showed a positively skewed distribution. Significance level was set at 5%.

## Results

### Baseline characteristics

Patients’ characteristics at diagnosis are summarized in Table 1. Eighty-six patients (15 men, 71 women) met the inclusion criteria. A microadenoma was noted in 45 patients (52%) and a macroadenoma in 41 (48%). The prevalence of microprolactinomas was not significantly greater in women than in men (56% vs. 33%, *p* = 0.16). There was a non-significant tendency towards lower age, lower BMI, and lower prevalence of headache in patients with microadenomas compared to patients with macroadenomas. Patients with microadenomas had significantly lower PRL levels than those with macroadenomas. There was a statistically non-significant tendency for an increased prevalence of gonadotrophic, thyrotrophic and corticotrophic insufficiency in macroadenomas compared to microadenomas. Prolactinoma extension to the medial carotid line (i.e., Knosp grade 1) was not noted in any patients with a microprolactinoma, and in 17 patients (41%) with macroprolactinomas (*p* < 0.001).

**Table 1:** Patient characteristics at baseline

Baseline Characteristics	Microadenoma		Macroadenoma		Total			*P* value
Number of patients, n (%)	45 (52)		41 (48)		86 (100)			
Age at diagnosis in years (mean ± SD)	32.9 ± 8.1		37.3 ± 13.4		35.0 ± 11.1			0.08
Women, n (%)	40 (90)		31 (76)		71 (83)			0.16
BMI (kg/m^2^ ± SD)	25.4 ± 5.8		27.5 ± 5.3		26.4 ± 5.6			0.20
Headache, n (%)	7 (16)		11 (29)		18 (22)			0.19
Affected pituitary axes, *n* (%)								
Gonadotropin deficiency	32 (71)		21 (88)		53 (77)			0.15
Secondary hypothyroidism	2 (4)		3 (9)		5 (6)			0.65
Secondary adrenal insufficiency	0 (0)		3 (4)		3 (4)			0.08
Knosp grade 1	0 (0)		17 (41)		17 (20)			** < 0.001**
Prolactin levels in μg/L (median; IQR)	130 (68–197)		303 (207–1100)		199 (94–458)			**0.01**

*BMI* body mass index, *n* numbers, *SD* standard deviation, *IQR* interquartile range

### Early postoperative remission rates

**Fig. 2:**
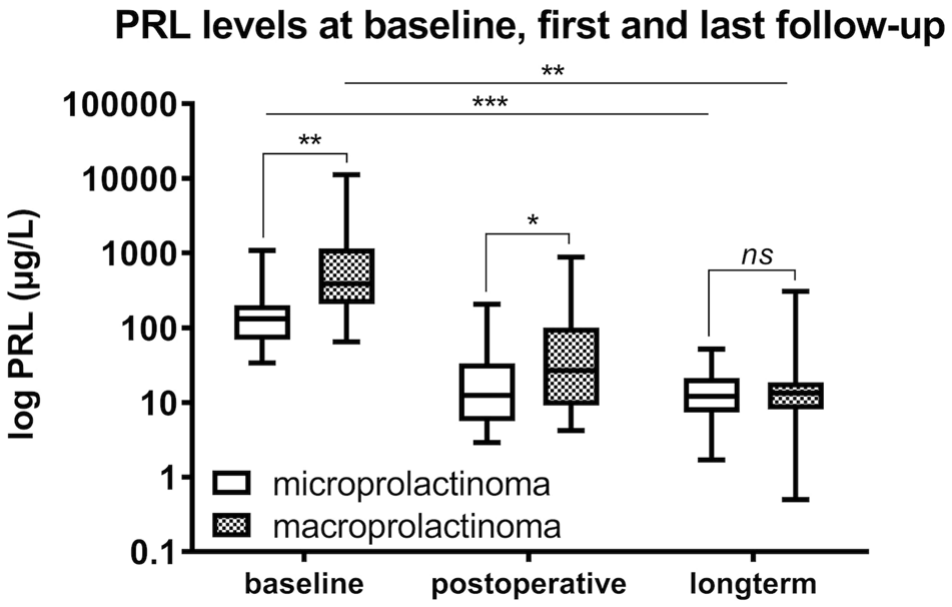
Impact of first-line surgery on PRL levels as a function of adenoma size. Differences in PRL levels before and after surgery in relation to adenoma size. Both baseline and postoperative PRL levels are significantly higher in patients with macroprolactinomas than those with microprolactinomas (*p* = 0.01 and *p* = 0.04, respectively), but not at long-term follow-up (*p* = 0.39). PRL levels significantly decreased in both cohorts compared to baseline, independent of the initial tumor size (i.e., microadenoma or macroadenoma). There is a significant difference between postoperative and long-term PRL values (*p* = 0.01 for microadenomas; *p* = 0.03 for macroadenomas, respectively). (***p < 0.001; **p < 0.01; *p < 0.05)

Immunohistological staining confirmed a prolactinoma in all patients. Serum PRL values decreased significantly in both cohorts, from 130 μg/L (IQR 68–197 μg/L) to 12 μg/L (IQR 6–28 μg/L), *p* < 0.001, in microadenomas and from 303 μg/L (IQR 207–1100 μg/L) to 28 μg/L (IQR 9–114 μg/L), *p* = 0.01, in macroadenomas (Fig. 2). Postoperative PRL values remained significantly higher in patients with macroprolactinomas than in those with microprolactinomas (*p* = 0.03). Postoperative normalization of PRL levels was obtained in 32 patients (76%) with microadenomas and in 18 patients (53%) with macroadenomas (*p* = 0.05). Among the subgroup of patients with macroprolactinomas only, remission was noted in 14 (74%) of them with a Knosp grade 0 prolactinoma, and in 4 (27%) with a Knosp grade 1 prolactinoma (*p* = 0.01).

Univariable analysis revealed that male sex, high preoperative PRL levels, Knosp grading (i.e., Knosp grade 1), and tumor size (i.e., macroadenoma) were related to early negative surgical results (i.e., PRL levels > 20 μg/L). No significant risk factors for early negative outcome were noted in the multivariable analysis (Table 2).

**Table 2:** Predictors of early negative outcome (postoperative PRL levels > 20 μg/L)

Predictive factors	Univariable analyses OR (95% CI)	*P* value	Multivariable analyses OR (95% CI)	*P* value
Age (years)	1.0 (1.0–1.1)	0.71		
Sex (male)	6.1 (1.7–22.4)	**0.01**	2.3 (0.4–15.0)	0.39
Headache (baseline)	1.0 (0.3–3.1)	0.99		
Hypopituitarism (baseline)	0.7 (0.2–1.9)	0.45		
Baseline BMI (kg/m^2^)	1.0 (0.9–1.1)	0.70		
PRL levels (baseline)	13.0 (3.2–52.5)	** < 0.001**	11.1 (2.1–59.4)	0.05
Knosp grading (Knosp grade 1)	8.4 (2.3–30.5)	**0.001**	3.3 (0.6–19.0)	0.17
Adenoma size (Macroadenoma)	2.8 (1.1–7.6)	**0.04**	1.8 (0.4–8.0)	0.41

*BMI* body mass index, *CI* confidence intervals, *DA* dopamine agonist, *OR* odds ratio, *PRL* prolactin

### Long-term remission rates

i) Surgery alone

Remission was achieved with surgery alone in 49 patients (59%), including 31 (72%) with a microprolactinoma and 18 (45%) with a macroprolactinoma (*p* = 0.02). With regard to macroprolactinomas only, surgery alone resulted in long-term remission in 15 patients (65%) with a Knosp grade 0 prolactinoma, compared to 3 patients (18%) with a Knosp grade 1 prolactinoma (*p* = 0.004). Thereby, recurrence-free intervals were not significantly longer in patients with a microadenoma (354 ± 29.7 months) than in those with a macroadenoma (339.3 ± 43.7 months); log-rank test, *p* = 0.50. However, the recurrence-free intervals were significantly shorter in patients with a Knosp grade 1 adenoma (110.5 ± 32.2 months) than in those with a Knosp grade 0 adenoma (365.4 ± 22.9 months; log-rank test, *p* < 0.001).

## ii) Multimodal treatment

**Fig. 3:**
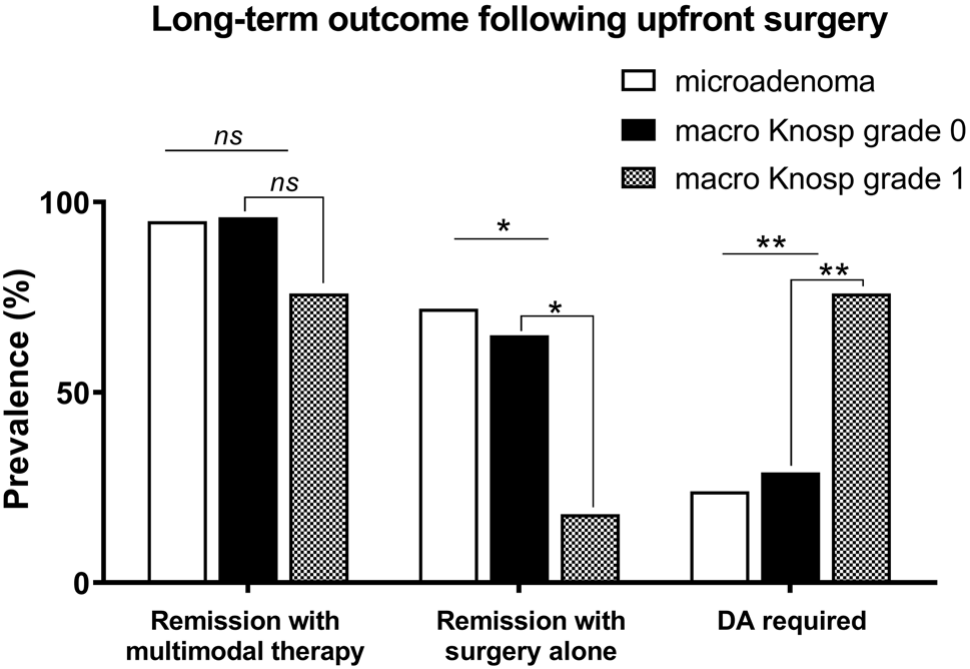
Long-term outcome following first-line surgery. Multimodal treatment (i.e., surgery ± DA) resulted in long-term control of hyperprolactinemia in 41 patients (95%) with a microprolactinoma vs. 35 patients (88%) with a macroprolactinoma (*p* = 0.25), namely in 22 macroadenomas (96%) of Knosp grade 0 vs. 13 (76%) with Knosp grade 1 (*p* = 0.14). Surgery alone resulted in long-term remission in 31 patients (72%) with a microprolactinoma vs. 18 patients (45%) with a macroprolactinoma (*p* = 0.02); namely in 15 (68%) patients with a macroadenoma Knosp grade 0 vs. 3 (18%) patients with a macroadenoma Knosp grade 1 (*p* = 0.004). For the long-term control of hyperprolactinemia, a significantly greater need for DA therapy was noted in patients with a macroprolactinomas (49%) than in patients with a microprolactinomas (24%, *p* = 0.03), and in macroprolactinomas Knosp grade 1 (76%) compared to macroprolactinomas Knosp grade 0 (29%, *p* = 0.004) (**p < 0.01; *p < 0.05)

**Fig. 4:**
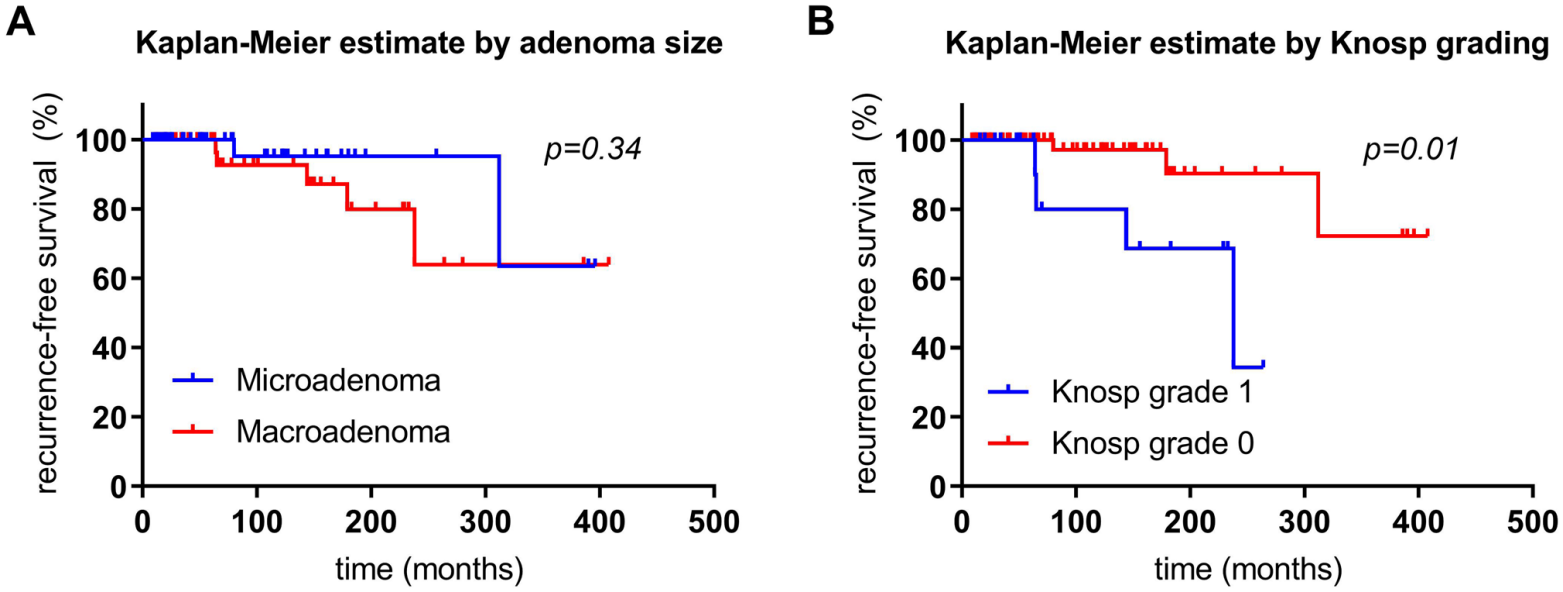
Kaplan–Meier estimation of recurrence-free intervals. **a** Recurrence-free intervals were not significantly shorter in patients with a microadenoma (354.3 ± 25.6 months) than in those with a macroadenoma (324.4 ± 33.2 months); log-rank test, *p* = 0.34. **b** However, recurrence-free intervals were significantly shorter in patients with a Knosp grade I prolactinoma (201.5 ± 25.2 months) than in those with a Knosp grade 0 prolactinoma (396.4 ± 22.5 months; log-rank test, *p* = 0.01)

Long-term remission was attained in 76 patients (92%) with multimodal treatment (i.e., surgery ± DA), namely in 41 patients (95%) with a microprolactinoma vs. 35 patients (88%) with a macroprolactinoma; *p* = 0.25; Fig. 3). Thereby, recurrence-free intervals were not significantly shorter in patients with a microadenoma (354.3 ± 25.6 months) than in those with a macroadenoma (324.4 ± 33.2 months); log-rank test, *p* = 0.34 (Fig. 4a). However, the recurrence-free intervals were significantly shorter in patients with a Knosp grade 1 prolactinoma (201.5 ± 25.2 months) than in those with Knosp grade 0 prolactinoma (396.4 ± 22.5 months; log-rank test, *p* = 0.01; Fig. 4b). With regard to macroprolactinomas only, multimodal treatment resulted in long-term remission in 22 patients (96%) with a Knosp grade 0 adenoma vs. 13 patients (76%) with a Knosp grade 1 adenoma (*p* = 0.14; Fig. 3).

### Long-term dependence on DAs

For the long-term control of hyperprolactinemia, a significantly greater need for persistent DA therapy was noted in 20 patients (49%) with a macroprolactinoma compared to 11 patients (24%) with a microprolactinoma (*p* = 0.03). In particular, as for macroprolactinomas, DA was required in 13 patients (76%) with Knosp grade 1 macroadenomas compared to 7 patients (29%) with Knosp grade 0 macroadenomas (*p* = 0.004; Fig. 3). Specifically, DAs were required in 11 patients (24%) with microadenomas, i.e. bromocriptine in 3 (6%) and cabergoline in 8 patients (18%), compared to 20 (49%) patients with a macroadenoma (i.e., bromocriptine in 8 (20%), and cabergoline in 12 (29%) patients (*p* = 0.03). Of the 31 (36%) patients receiving DAs at last follow-up, 11 (13%) patients received bromocriptine, and 20 (23%) patients received cabergoline. Daily doses at last follow-up were 5.0 ± 2.6 mg for bromocriptine, 61.4 ± 19.3 µg for quinagolide, and 0.53 ± 0.21 mg for cabergoline Table 3).

The risk factors for long-term DA dependence are summarized in Table 4. Significant risk factors in the univariable analysis included male sex and Knosp grade 1 prolactinomas. Multivariable Cox regression analyses revealed Knosp grading but not adenoma size (i.e., macroadenoma) as an independent risk factor for dependence on DAs. Eight patients (9%) successfully quit DA therapy during a median follow-up of 140 months.

### Characteristics at last follow-up

Patients’ characteristics at last follow-up are summarized in Table 3. The median follow-up period was 80 (13–408) months and did not differ between the two cohorts. Baseline PRL levels were significantly higher in patients with long-term dependence on DAs than in those without; 284 µg/L (IQR 200–1000 µg/L) vs. 136 µg/L (IQR 75–234 µg/L), *p* = 0.04.

**Table 3:** Patient characteristics at last follow-up

Characteristics at last follow-up	Microadenoma	Macroadenoma	Total	P value
Follow-up time in months (median, range)	79 (13–396)	97 (13–408)	80 (13–408)	0.3
BMI (kg/m2 ± SD)	24.7 ± 5.7	27.7 ± 5.0	26.1 ± 5.6	**0.02**
Headache, n (%)	1 (2)	1 (2)	2 (2)	0.99
Affected pituitary axes, n (%)				
Gonadotropin deficiency	5 (19)	8 (38)	13 (27)	0.19
Secondary hypothyroidism	2 (5)	6 (15)	8 (9)	0.15
Secondary adrenal insufficiency	0 (0)	3 (8)	3 (4)	0.1
Prolactin levels in μg/L (median; IQR)	11.9 (7.3–21.0)	13.8 (8.2–20.4)	12.7 (7.5–20.9)	0.29
Prolactin levels normalized				
Multimodal treatment	41 (95)	35 (88)	76 (92)	0.25
Surgery alone	31 (72)	18 (45)	49 (59)	**0.02**
Dopamine agonists required	11 (24)	20 (49)	31 (36)	**0.03**

*BMI* body mass index, *n* numbers, *SD* standard deviation, *IQR* interquartile range

**Table 4:** Predictors of long-term dependence on dopamine agonists

Predictors of long-term dopamine agonist dependence	Univariable analyses HR (95% CI)	*P* value	Multivariable analyses HR (95% CI)	*P* value
Age (years)	1.0 (1.0–1.0)	0.65		
Sex (male)	2.6 (1.2–5.9)	**0.02**	1.5 (0.6–4.2)	0.39
Headache (baseline)	2.2 (0.9–5.3)	0.07		
Hypopituitarism (baseline)	0.6 (0.3–1.5)	0.29		
Baseline BMI (kg/m^2^)	1.0 (0.9–1.1)	0.45		
PRL levels (baseline)	1.6 (0.9–3.0)	0.10		
Knosp grading (Knosp grade 1)	2.7 (1.3–5.6)	**0.01**	2.2 (1.0–5.4)	**0.03**
Adenoma size (Macroadenoma)	1.5 (0.7–3.2)	0.28		

*BMI* body mass index, *CI* confidence intervals, *DA* dopamine agonist, *HR* hazard ratio, *PRL* prolactin

## i) Surgery alone

Compared to postoperative values, PRL levels remained stable both in the microadenoma and the macroadenoma cohorts, namely from 12 µg/L (IQR 7–20 µg/L) to 7 μg/L (IQR 5–14 μg/L), p = 0.99 (microadenoma cohort), and from 15 µg/L (IQR 10–22 µg/L) to 12 μg/L (IQR 7–45 μg/L), *p* = 0.13, in the macroadenoma cohort, respectively. At last follow-up, serum PRL levels in patients with a microadenoma were not significantly different from those in patients with a macroadenoma (*p* = 0.41). Likewise, PRL levels were not significantly different in patients with Knosp grade 1 prolactinomas compared to those with Knosp grade 0 prolactinomas (*p* = 0.25). The number of patients with headache dropped from 11 (20%) to 2 (4%) (*p* = 0.01), both in patients with microprolactinomas, though not significantly (12% vs. 3%, *p* = 0.19) and in patients with macroprolactinomas (33% vs. 5%, *p* = 0.05). There was a significant decrease in the prevalence of hypogonadism (*p* < 0.001), both in patients with microprolactinomas (71% vs. 10%, *p* < 0.001) and macroprolactinomas (92% vs. 25%, *p* = 0.003), with no significant difference in the prevalence of hypogonadism at long-term follow-up between the two cohorts (*p* = 0.55). At the final follow-up, gonadotropic, thyrotropic and corticotropic insufficiency were not significantly different between the two groups.

## ii) Multimodal treatment

Compared to postoperative values, PRL levels remained stable in the microadenoma cohort and decreased in the macroadenoma cohort, namely from 12 μg/L (IQR 6–28 μg/L) to 12 µg/L (IQR 7–21 µg/L), *p* = 0.20 (microadenoma cohort), and from 28 μg/L (IQR 9–114 μg/L) to 14 µg/L (IQR 8–20 µg/L), *p* = 0.03, in the macroadenoma cohort, respectively. At last follow-up, serum PRL levels in patients with microadenomas were not significantly different from those in patients with macroadenomas (Fig. 2). Likewise, PRL levels were not significantly different in patients with Knosp grade 1 prolactinomas compared to those with Knosp grade 0 prolactinomas (*p* = 0.24). The number of patients with headache dropped from 18 (22%) to 2 (2%) (*p* < 0.001), which was significant both in patients with a macroprolactinoma (29% vs. 2%, *p* = 0.001) and a microprolactinoma (16% vs. 2%, *p* = 0.03). There was a significant decrease in the prevalence of hypogonadism, both in patients with microprolactinomas (93% vs. 19%, *p* < 0.001) and macroprolactinomas (88% vs. 38%, *p* = 0.001), with no significant difference in the prevalence of hypogonadism between the two cohorts over the long term. At last follow-up, gonadotropic, thyrotropic and corticotropic insufficiency were not significantly different between the two groups.

### Morbidity and mortality

There was no mortality in patients with a microadenoma or a macroadenoma. Surgical complications consisted of transient rhinoliquorrhea (3%) requiring transsphenoidal revision by autologous fat graft and dural patching in two patients following surgery on a Knosp grade 1 macroprolactinoma. Transient syndrome of inappropriate secretion of antidiuretic hormone (10%) and diabetes insipidus (13%), as well as slight upper bitemporal quadrantal hemianopsia, was seen in one patient. We noted no vascular injuries, meningitis or abscesses.

Compared to baseline, new thyrotrophic insufficiency after surgery was noted in 6 patients (7%), with 5 (6%) harboring a macroprolactinoma. New corticotrophic insufficiency was noted in 2 patients (2%), with 1 patient (1%) harboring a macroprolactinoma.

## Discussion

The present analysis of the largest series reported to date with a surgery-first approach reveals that over the long term (7 years) (i) upfront surgery resulted in a high likelihood of avoiding DA therapy in patients with microprolactinomas; (ii) persistent hyperprolactinemia and the need for adjuvant DA therapy is significantly greater in patients with a macroprolactinoma, especially in those with a Knosp grade 1 adenoma; and (iii) no significant short-term or long-term morbidity or mortality could be documented.

TSS and DA represent effective treatment options for prolactinomas [50]. However, long-term treatment with DAs is often required, and can lead to potential adverse effects, in particular the recently documented personality changes associated with DAs, [24] lack of compliance, and limited convenience for patients [21, 27, 51].

In recent years, the scale of indications has tipped towards TSS due to its favorable outcome and the potential adverse effects of DAs over the long term [52]. This approach is reinforced by two recent meta-analyses confirming that disease remission can be achieved with surgery in the majority of patients [7, 8]. As a result, choosing to perform surgical treatment as a first-line approach might be beneficial given the potential for adenoma fibrosis associated with DA therapy. Fibrosis has been shown to hamper outcome, [53, 54] but results remain controversial [5]. Consequently, the PRolaCT trial started recruiting participants with the goal of investigating whether TSS for microprolactinoma and macroprolactinoma resection is superior to standard care as a first-line approach or a second-line treatment (NCT 04,107,480).

As for macroprolactinomas, they often present with higher prolactin levels and a tendency to extend to the cavernous sinus, as documented in the current study with 41% of patients with a Knosp grade 1 macroadenoma. Consequently, upfront surgery did not result in long-term remission in a substantial number of patients, and there was a need for long-term DA therapy after surgery in about half of them. Likewise, Donegan et al. reported persistent dependence on DAs in 66% of their patients [55]. The slightly higher need for continuing DA therapy might be due to their inclusion of patients with prior DA therapy (in contrast to the present study) in whom perivascular tumor fibrosis may hamper complete adenoma resection, as mentioned above [53, 54, 56]. All the same, although recurrence-free intervals did not differ significantly with regard to adenoma size, patients with adenoma infiltration had significantly shorter recurrence-free intervals than those without. It is conceivable that the smaller sample size of patients with macroadenomas conceals a true effect [57].

Considering these factors, the current study indicates that a surgery-first approach should not be considered in patients with macroprolactinomas.

Invasive macroprolactinomas in particular represent a therapeutic challenge. We noted that surgery alone resulted in long-term remission in 65% of patients with Knosp grade 0 macroadenomas, as opposed to only 18% with Knosp grade 1 adenomas (*p* = 0.004). In keeping with the finding that the Knosp grading was an independent predictor of long-term dependence on DAs, upfront surgery cannot be recommended in patients with a Knosp grade 1 prolactinoma.

Pituitary tumors in general have a considerable impact on patients’ functional status [58]. Thus, quality of life is impaired over the long-term in many patients with pituitary adenomas compared to the general population [59, 60]. Recently, it has been demonstrated that healthcare utilization and costs of patients treated for a prolactinoma are mainly associated with health-related quality of life.[61] Interestingly, tumor size and prolactinoma treatment were however not significantly associated with healthcare utilization [61]. In addition, treatment costs were not significantly higher for patients with a macroprolactinoma, except in terms of medication costs [61]. With regard to the treatment strategy being chosen, transsphenoidal surgical resection of microprolactinomas has been shown to be more cost-effective than life-long medical therapy in young patients with a life expectancy greater than 10 years [62]. However, these results require that surgery be performed at high-volume centers by experienced pituitary surgeons with low complication rates. Therefore, dependence on DAs in some patients with a macroprolactinoma should also be considered in relation to the potential risks of surgery. Although morbidity, mortality and rates of new endocrinopathies (3% and 0%, and 5%, respectively) in our study are in line with previous findings, [63] they are not nonexistent. In contrast, DAs can be easily administered and monitored and are usually well tolerated. In addition, they have well-known anti-secretory and anti-proliferative effects [64]. With regard to the low need for persistent DAs over the long term in our cohort of patients with first-line surgery—in particular those with microprolactinomas—indiscriminate prescription of DAs at diagnosis without the support of interdisciplinary consensus findings or careful evaluation by MRI cannot be advised. Even if side effects are only infrequently encountered, they can still have severe psychosocial implications including debt, in particular in the case of DA-induced personality changes (gambling, hypersexuality, compulsive shopping), [21, 24] which are usually not immediately reported by patients as they feel intense shame. Although DA’s have not been reported to induce systemic fibrosis at low doses, [65–67] Ono and colleagues reported that up to 18% of patients needed persistent high-dose cabergoline treatment to normalize hyperprolactinemia, irrespective of tumor size [68]. In this regard, cumulative doses of DA might account for long-term adverse effects, [23, 69, 70] and new concerns about long-term safety of DAs have emerged over time [21, 23, 24, 52]. Of note, in contrast to initial studies reporting that many patients treated with cabergoline remained in remission after drug withdrawal, [47] later reports have described early recurrence of hyperprolactinemia following discontinuation of DAs, particularly in patients with macroprolactinomas [71–73]. These results are in line with a recent meta-analysis showing that long-term remission was lower after DA withdrawal (34%) than after transsphenoidal surgery (64%), [8] while a previous meta-analysis reported even lower (21%) long-term remission rates after DA withdrawal [14].

### Study limitations

Follow-up at < 24 months in a few patients may have interfered with the results of long-term dependence on DAs, as our treatment regime consisted of tapering medications 24 months after initiation of the medical therapy if PRL levels had normalized and/or adenoma reduction of > 50% was attained. Because follow-up continued for such a long time, data on pituitary insufficiency was missing for some patients. This included gonadotrophic insufficiency in 38 patients (44%), thyrotrophic insufficiency in 1 patient (1%) and corticotrophin insufficiency in 2 patients (2%). Likewise, long-term data on patients’ BMI (kg/m^2^) was missing for 13 patients (15%).

Operations were performed by three different neurosurgeons, although mainly by one surgeon. In general, surgeons with higher levels of experience are associated with better outcomes in numerous studies [74, 75].

Values for individual adenoma size measurements and information about sphenoid sinus invasion in this patient cohort are missing, and allocation into groups (i.e., microadenoma and macroadenoma) doesn’t allow further subanalyses.

## Conclusion

First-line surgery in patients with microprolactinomas or macroprolactinomas of Knosp grade 0 resulted in a good chance of non-dependency on DA therapy. However, in patients with prolactinomas Knosp grade 1, first-line surgery cannot be recommended, as adjuvant DA therapy after surgery is required in the majority over the long term.

## Abbreviations

DA: Dopamine agonist
MRI: Magnetic resonance imaging
PRL: Prolactin
TSS: Transsphenoidal surgery
